# BMP9 induces osteogenesis and adipogenesis in the immortalized human cranial suture progenitors from the patent sutures of craniosynostosis patients

**DOI:** 10.1111/jcmm.13193

**Published:** 2017-05-04

**Authors:** Dongzhe Song, Fugui Zhang, Russell R. Reid, Jixing Ye, Qiang Wei, Junyi Liao, Yulong Zou, Jiaming Fan, Chao Ma, Xue Hu, Xiangyang Qu, Liqun Chen, Li Li, Yichun Yu, Xinyi Yu, Zhicai Zhang, Chen Zhao, Zongyue Zeng, Ruyi Zhang, Shujuan Yan, Tingting Wu, Xingye Wu, Yi Shu, Jiayan Lei, Yasha Li, Wenwen Zhang, Jia Wang, Michael J. Lee, Jennifer Moriatis Wolf, Dingming Huang, Tong‐Chuan He

**Affiliations:** ^1^ State Key Laboratory of Oral Diseases National Clinical Research Center for Oral Diseases West China Hospital of Stomatology Sichuan University Chengdu China; ^2^ Molecular Oncology Laboratory Department of Orthopaedic Surgery and Rehabilitation Medicine The University of Chicago Medical Center Chicago IL USA; ^3^ Ministry of Education Key Laboratory of Diagnostic Medicine and the Affiliated Hospitals of Chongqing Medical University Chongqing China; ^4^ Department of Surgery Section of Plastic Surgery The University of Chicago Medical Center Chicago IL USA; ^5^ Department of Biomedical Engineering School of Bioengineering Chongqing University Chongqing China; ^6^ Departments of Neurosurgery and Otolaryngology‐Head & Neck Surgery the Affiliated Zhongnan Hospital of Wuhan University Wuhan China; ^7^ Department of Emergency Medicine Beijing Hospital Beijing China; ^8^ Department of Orthopaedic Surgery Union Hospital of Tongji Medical College Huazhong University of Science & Technology Wuhan China; ^9^ Department of Laboratory Medicine and Clinical Diagnostics the Affiliated Yantai Hospital Binzhou Medical University Yantai China

**Keywords:** cranial suture, craniosynostosis, BMP9, suture‐derived stem cells, osteogenic differentiation, adipogenesis, cell immortalization

## Abstract

The cranial suture complex is a heterogeneous tissue consisting of osteogenic progenitor cells and mesenchymal stem cells (MSCs) from bone marrow and suture mesenchyme. The fusion of cranial sutures is a highly coordinated and tightly regulated process during development. Craniosynostosis is a congenital malformation caused by premature fusion of cranial sutures. While the progenitor cells derived from the cranial suture complex should prove valuable for studying the molecular mechanisms underlying suture development and pathogenic premature suture fusion, primary human cranial suture progenitors (SuPs) have limited life span and gradually lose osteoblastic ability over passages. To overcome technical challenges in maintaining sufficient and long‐term culture of SuPs for suture biology studies, we establish and characterize the reversibly immortalized human cranial suture progenitors (iSuPs). Using a reversible immortalization system expressing SV40 T flanked with FRT sites, we demonstrate that primary human suture progenitor cells derived from the patent sutures of craniosynostosis patients can be efficiently immortalized. The iSuPs maintain long‐term proliferative activity, express most of the consensus MSC markers and can differentiate into osteogenic and adipogenic lineages upon BMP9 stimulation *in vitro* and *in vivo*. The removal of SV40 T antigen by FLP recombinase results in a decrease in cell proliferation and an increase in the endogenous osteogenic and adipogenic capability in the iSuPs. Therefore, the iSuPs should be a valuable resource to study suture development, intramembranous ossification and the pathogenesis of craniosynostosis, as well as to explore cranial bone tissue engineering.

## Introduction

Cranial bones are flat bones and formed through intramembranous ossification, which is a process of the condensation and proliferation of MSCs and subsequent differentiation into osteoblasts and osteocytes 1, 2, 3, 4, 5. Cranial vault sutures are fibrous tissues uniting the adjacent osteogenic fronts of two calvaria. During craniofacial development, they serve as the major sites of bone growth and the suture mesenchyme has been reported to serve as the niche of skeletal stem cells 6. Functionally, sutures need to remain in an unfused state from infancy through early adulthood, and in the meanwhile, the cells in the middle of suture mesenchyme remain undifferentiated, yet allow bone to be formed at the edges of the bone fronts in response to external stimuli and allow the skull to change shape and grow during development 3, 4, 5, 10, 11, 12. Thus, patency and fusion of cranial sutures are highly coordinated and tightly regulated processes during development.

Craniosynostosis is a common congenital malformation and is the premature fusion of one or more of the cranial sutures, occurring approximately in 1 of 2000–2500 live births 1, 3, 4, 5, 10, 11, 12. In patients with craniosynostosis, the normal growth of the skull is restricted by premature ossification of the cranial sutures leading to numerous morphologic abnormalities, functional deficiencies and potential developmental delay 11, 13. Currently, patients with craniosynostosis are treated surgically soon after diagnosis to reshape the skull and attempt to avoid downstream problems with elevated intracranial pressure 12, 14, although there have been attempts to explore molecular therapies targeting the affected pathways in the past few years 9, 15, 16, 17. Craniosynostosis is clinically categorized as non‐syndromic or syndromic in nature. Mechanistically, mutations in FGFRs, Msx2 and Twist are associated with the most common syndromes that manifest craniosynostosis 10, 12, 14. Members of the BMP and TGFβ superfamily are also implicated in craniosynostosis 15, 18. Nonetheless, the molecular biology underlying suture development and pathogenic process of non‐syndromic craniosynostosis remain to be fully understood.

The cranial suture complex is a heterogeneous tissue consisting of osteogenic progenitor cells and MSCs from bone marrow and suture mesenchyme 6, 19, 20. MSCs are multipotent stem cells that are able to differentiate into multiple lineages, including osteogenic, chondrogenic and adipogenic lineages, and have been isolated from numerous tissues including bone marrow, periosteum, liver and adipose 21, 22, 23, 24. MSCs can be isolated from cranial bone marrow and cranial suture mesenchyme 6, 8, 20, 25, as well as human primary cranial suture cells 19, 26, 27, 28, 29, 30.

Early studies indicated that human cranial suture cells possess similar characteristics of pre‐osteoblastic progenitor cells 28, 29, 30, 31. Therefore, the progenitor cells derived from cranial suture complex may serve as a valuable resource for studying the molecular mechanisms underlying suture development and pathogenic premature suture fusion. Although mouse suture MSCs were shown to retain relatively long‐term self‐renewing, clonal expanding and differentiating abilities under special conditions 8, the human primary cranial suture cells usually have limited life span *in vitro* and may lose osteoblastic ability after certain passages 19. Therefore, it is highly desirable to establish stable human cranial SuPs, which ideally possess long‐term proliferative capability while retaining osteogenic potential.

Here, we established the reversibly iSuPs by introducing SV40 T antigen into the primary SuPs derived from unfused cranial sutures of craniosynostosis patients. We demonstrated that the iSuPs maintain long‐term proliferative activity, express most of the consensus MSC markers and possess osteogenic and adipogenic potential when stimulated with the potent osteogenic and adipogenic factor BMP9 32, 33, 34, 35. The immortalization of iSuPs can be reversed by the removal of SV40 T antigen. Therefore, the iSuPs should be a valuable resource to study suture development and pathogenesis of premature suture fusion in craniosynostosis, as well as a potential therapeutic agent for cranial bone tissue engineering.

## Materials and methods

### Cell culture and chemicals

HEK‐293 (from ATCC, Manassas, VA, USA) and its derivative line 293pTP cells were maintained in the completed Dulbecco's modified Eagle medium (DMEM) as 36, 37, 38, 39. Unless indicated otherwise, all chemicals were purchased from Sigma‐Aldrich (St. Louis, MO, USA) or Thermo Fisher Scientific (Waltham, MA, USA).

### Isolation of cranial SuPs from the unfused (patent) coronal sutures of craniosynostosis patients

The use of human cranial suture samples was approved by the institutional review board. The informed consent forms were signed by the guardians of the affected children according to the approved guidelines by the Institutional Review Board. The unfused (patent) coronal sutures were retrieved from three male patients, aged 15–17 months and undergoing cranial vault reconstruction for craniosynostosis at the Comer Children's Hospital of The University of Chicago Medicine. The suture samples consisted of suture mesenchyme plus approximately 5 mm of bone on either side and were placed in Ringers solution until processed in the laboratory.

The primary cranial SuPs were grown from human suture samples 19. Briefly, the dissociated suture samples were rinsed with cold sterile PBS with 1% penicillin/streptomycin solution, and minced into 1.0 mm fragments, followed by incubation in 0.25% trypsin/1 mM EDTA with gentle agitations at 37°C for 30 min. Ten milliliters of complete DMEM was added to inactivate trypsin. The digested cell/tissue mixture was transferred to 100‐mm cell culture dishes and incubated in a humidified atmosphere of 5% CO_2_ maintained at 37°C. After approximately 10–14 days, cells grew to 80% confluency at that point and were passaged to 25‐cm^2^ flasks containing 8 ml of complete DMEM for experimentation. While primary SuPs were stored in liquid nitrogen tanks, passages #2 to #5 were used in this study.

### Establishment of reversibly immortalized cranial suture progenitors (iSuPs)

The use of the retroviral vector SSR #41 or SSR#69 to express SV40 T antigen flanked with the FRT or loxP sites has been previously described 40, 41, 42, 43, 44, 45, 46, 47, 48. Briefly, the SSR #41 vector and pCL‐Ampho packaging vector were cotransfected into HEK‐293 cells to produce the packaged retrovirus. The stably immortalized cranial suture progenitors were established by infecting the primary SuPs with retrovirus and selecting with hygromycin B (0.3 mg/ml) for 5–7 days, designated as iSuPs.

### Recombinant adenoviruses expressing BMP9, Flippase (FLP) and Green Fluorescent Protein (GFP)

Recombinant adenoviruses were generated using the AdEasy technology as previously described 49, 50. The coding regions of human BMP9, FLP recombinase and GFP were PCR amplified and cloned into an adenoviral shuttle vector and subsequently used to generate recombinant adenoviruses in HEK‐293 or 293pTP cells 39. The resulting adenoviruses were designated as Ad‐BMP9 and Ad‐FLP, both of which also express GFP as the marker for monitoring infection efficiency. Analogous adenovirus expressing only GFP (Ad‐GFP) was used as a control 51, 52. In order to enhance transgene transduction efficiency, polybrene (8 μg/ml) was added to the culture medium for all adenovirus infections 53.

### Crystal violet assay

Subconfluent cells were seeded in 35‐mm cell culture dishes and infected with the Ad‐FLP or Ad‐GFP adenovirus. The infected cells were subjected to crystal violet staining at the indicated time‐points. Macrographic staining images were recorded for the stained dishes. For quantitative measurement, the stained cells were dissolved in 10% acetic acid at room temperature with agitation and optical density was measured at 570~590 nm 51, 54.

### WST‐1 cell proliferation assay

Exponentially growing cells were infected with appropriate adenoviruses and/or plated into 96‐well culture plates at 20% confluence. Unseeded wells were utilized as background controls. At the indicated time‐points, the premixed WST‐1 (BD Clontech, Mountain View, CA, USA) was added to each well and incubated at the 37°C CO_2_ incubator for 3 hrs. The plates were subjected to a microtitre plate reader to obtain absorbance reading at 450 nm. The obtained A_450 nm_ values were subjected to background reading subtractions. Each assay condition was performed in triplicate 36, 55.

### Immunofluorescence staining

Immunofluorescence staining was performed 43, 51. Briefly, cells were seeded in 24‐well plates overnight, fixed with 4% paraformaldehyde, permeabilized with 1% NP‐40 and blocked with 10% donkey serum (Jackson Immuno‐Research Laboratories, West Grove, PA, USA), followed by incubating with CD73, CD105/endoglin, CD90/Thy‐1, CD166/ALCAM, BMPR‐II, CD117/c‐kit or CD29/Integrin β1 antibody (Santa Cruz Biotechnology, Dallas, TX, USA) for 1 hr at room temperature, as previously reported 43, 56. After being washed, cells were incubated with Texas Red or FITC labelled secondary antibody (Jackson ImmunoResearch Laboratories) for 30 min. Cell nuclei were counterstained with DAPI. Stains without primary antibodies were used as negative controls. Fluorescence images were recorded under an inverted fluorescence microscope.

### RNA isolation and Touchdown qPCR (TqPCR) analysis

Subconfluent cells were seeded in 60‐mm dishes in a complete DMEM with different treatments. Total RNA was isolated using TRIzol Reagents (Invitrogen, Carlsbad, CA, USA) according to the manufacturer's instructions. The cDNA synthesis was carried out using hexamer and M‐MuLV Reverse Transcriptase (New England Biolabs, Ipswich, MA, USA). The cDNA products were diluted 10‐ to 50‐fold and used as PCR templates. PCR primers (Table S1) were designed using Primer3 Plus 57 (approximately 100–200 bp). The TqPCR program was carried out as follows: 95°C × 3 sec. for one cycle; 95°C × 20 sec., 66°C × 10 sec., for 4 cycles by decreasing 3°C per cycle; 95°C × 20 sec., 55°C × 10 sec., 70°C × 1 sec., followed by plate read, for 40 cycles 58. GAPDH was used as a reference gene. Each assay condition was performed in triplicate.

### Alkaline phosphatase (ALP) activity assay

ALP activity was assessed quantitatively with a modified assay using the Great Escape SEAP Chemiluminescence assay kit (BD Clontech) and qualitatively with histochemical staining assay (using a mixture of 0.1 mg/ml naphthol AS‐MX phosphate and 0.6 mg/ml Fast Blue BB salt) 46, 59. Each assay condition was performed in triplicate, and the results were repeated in at least three independent experiments.

### Matrix mineralization assay (Alizarin Red S staining)

The iSuPs were seeded in 24‐well culture plates, infected with Ad‐BMP9 or Ad‐GFP and cultured in the presence of ascorbic acid (50 mg/ml) and β‐glycerophosphate (10 mM). At 14 days post‐infection, mineralized matrix nodules were stained for calcium precipitation using Alizarin Red S staining 32, 60. Briefly, cells were fixed with 2.5% glutaraldehyde at room temperature for 10 min. and washed with PBS (pH adjusted to 4.2), and fixed cells were left in 37°C incubator with 2% Alizarin Red S for 10 min., while staining was monitored under microscope every 2–5 min., followed by extensively rinsing with PBS. The staining of calcium mineral deposits was recorded under a bright field microscope. Each assay condition was performed in triplicate.

### Oil Red O staining assay

Exponentially growing cells were plated onto 24‐well culture plates and infected with Ad‐BMP9 or Ad‐GFP. Oil Red O staining was performed at 10 days post‐infection as previously described 35, 61. Briefly, cells were fixed with 10% formalin at room temperature for 10 min., followed by gentle washing with PBS. The fixed cells were stained with freshly prepared Oil Red O solution (six parts saturated Oil Red O dye in isopropanol plus four parts water) for 60 min. at room temperature, followed by washing with PBS. The staining of lipid droplets was recorded under a bright field microscope. Each assay condition was performed in triplicate.

### Subcutaneous implantation of BMP9‐transduced iSuPs for ectopic bone formation and adipogenesis

All animal care and use in this study followed the approved protocol by the Institutional Animal Care and Use Committee. Stem cell‐mediated ectopic bone formation was performed 59, 62. Briefly, iSuPs were infected with Ad‐BMP9 or Ad‐GFP for 24 hrs, harvested and resuspended in sterile PBS for subcutaneous injection (at 2 × 10^6^ cells/injection, 4 × 10^6^ cells/injection, and 6 × 10^6^ cells/injection; eight injections/dose/group; four injections/mouse) into the flanks of athymic nude (nu/nu) mice (female, 5–6 weeks old; Harlan Research Laboratories/ENVIGO, Indianapolis, IN, USA). At 4 weeks after implantation, the mice were killed, and the implantation sites were retrieved for micro‐CT imaging and histologic evaluations.

### Micro‐computed tomography (μCT) analysis

The retrieved specimens were fixed and imaged using the μCT component of the GE triumph (GE Healthcare, Piscataway, NJ, USA) trimodality preclinical imaging system. All image data analyses were performed using Amira 5.3 (Visage Imaging, Inc., San Diego, CA, USA), and 3D volumetric data were determined 61, 63.

### H&E staining and trichrome staining

After being imaged, the retrieved tissues were fixed, decalcified in 10% buffered formalin and embedded in paraffin. Serial sections of the embedded specimens were stained with haematoxylin and eosin (H & E). Trichrome stains were carried out 64, 65.

### Statistical analysis

All quantitative studies were carried out in triplicate and/or performed in three independent batches. Microsoft Excel program (Redmond, WA, USA) was carried out to calculate standard deviation (S.D.). Statistically significant differences between samples were determined by one‐way analysis of variance and the two‐tailed Student's *t*‐test. A value of *P* < 0.05 was considered statistically significant when one comparison was being made.

## Results

### Establishment of immortalized human cranial suture stem cells (iSuPs), which exhibit long‐term high proliferative activity

**Figure 1:**
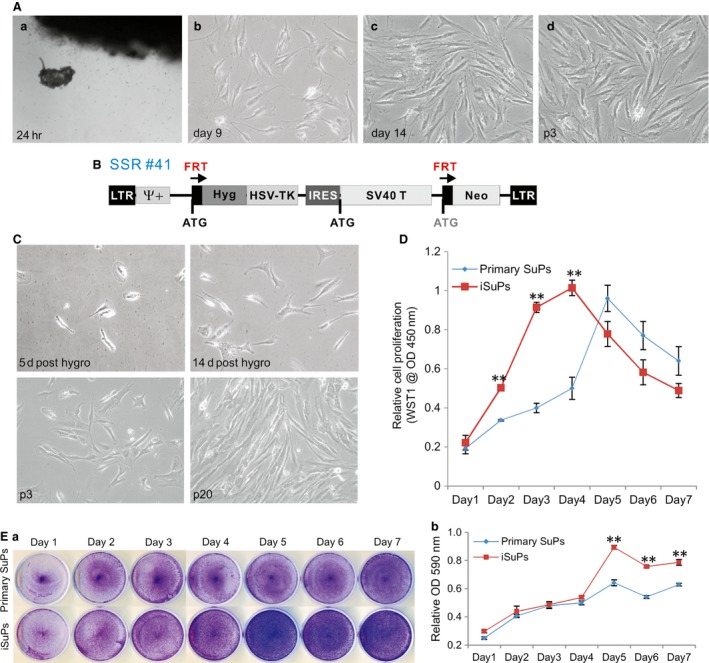
Immortalization of human cranial suture progenitor cells (iSuPs) derived from the patent suture tissues from craniosynostosis patients. (**A**) Primary suture progenitor cells (SuPs) were isolated from the freshly harvested cranial sutures and cultured in complete DMEM medium (*a*). Morphology of the recovered primary cells was recorded at day 9 (*b*) and day 14 (*c*) after plating, as well as at passage 3 (p3) (*d*). (**B**) Schematic representation of the reversible immortalization vector SSR #41. This retroviral vector contains the hygromycin and SV40 T antigen expression cassette flanked with FRT sites and can be removed by the Flippase (FLP) recombinase. (**C**) Establishment of iSuPs. The primary SuP cells were infected with packaged SSR #41 and selected in hygromycin‐containing medium for 5 days. Survived cells were observed at day 5 and day 14 post‐selection. The iSuPs were seeded at low density and passed consecutively for 3 (p3) or 20 passages (p20). Representative images are shown. (**D**) Cell proliferation evaluated by WST‐1 assay. The same number of primary SuPs (passage 2) and iSuPs was seeded at a low density. WST‐1 substrate was added to the cell culture and assessed for A_450 nm_ readings at the indicated time‐points. Assays were performed in triplicate. ‘**’*P* < 0.001. (**E**) Cell proliferation assessed by crystal violet staining assay. The same number of primary SuPs and iSuPs was plated with a low density and fixed for crystal violet staining at the indicated time‐points (*a*). The stained cells were dissolved for OD reading and quantitatively determined at A_590 nm_ (*b*). The assays were performed in three independent batches of experiments. Representative results are shown. ‘**’*P* < 0.001.

Although human cranial suture cells have been used rather extensively, their biological features are not thoroughly characterized 6, 19, 26. While readily available, it is time‐consuming to isolate and culture primary cranial suture cells. More importantly, it is challenging to obtain human clinical specimens of cranial sutures. Here, we sought to establish reversibly immortalized human suture progenitor cells (iSuPs) and to investigate their osteogenic and adipogenic differentiation capability. We isolated the primary cranial progenitor cell (SuPs) from the patent cranial sutures of three craniosynostosis patients 19, 42, 43. After 24 hrs, fibroblast‐like primary cells were observed migrating outwards from the minced fragments of suture tissues (Fig. 1A, panel a). Primary SuPs grew well in culture, albeit slowly, and the morphology of primary SuPs was shown at day 9 and day 14 (Fig. 1A, panels b and c), as well as at passage 3 (Fig. 1A, panel d).

The recovered and subconfluent primary SuPs were subjected to immortalization utilizing the retroviral vector SSR#41 that contains the hygromycin and SV40 T antigen expression cassette flanked with FRT sites (Fig. 1B) 40. We previously used this system to successfully obtain a large panel of reversibly immortalized progenitor cells 42, 43, 45, 46. After hygromycin selection, colonies that formed by the surviving cells were observed at as early as at 5 days after selection (Fig. 1C, panel a) and the colonies became more obvious at 14 days after selection (Fig. 1C, panel b). The iSuPs grew more rapidly and maintained a high proliferation rate after 20 passages (Fig. 1C, panels c & d). In fact, the iSuPs have been passed consecutively for more than 60 generations thus far and proliferate well. Thus, these results indicate that we successfully immortalized SuPs.

We subsequently compared the proliferative activities between primary SuPs and iSuPs. WST‐1 cell proliferation assay showed that iSuPs exhibited a higher proliferation rate than that of SuPs, especially within the first 4 days (Fig. 1D). Likewise, crystal violet staining assay indicated that iSuPs reached confluence at as early as day 5, while primary SuPs reached confluence at day 7 when both started with the same cell density (Fig. 1E, panel a). Quantitative assessment of the stained cells confirmed the staining results that iSuPs had significantly higher cell staining after day 5 than that of the SuPs (Fig. 1E, panel b). The above results demonstrate that the iSuPs can be stably maintained in culture and exhibit a high proliferative rate.

### The iSuPs express most of the MSC markers

**Figure 2:**
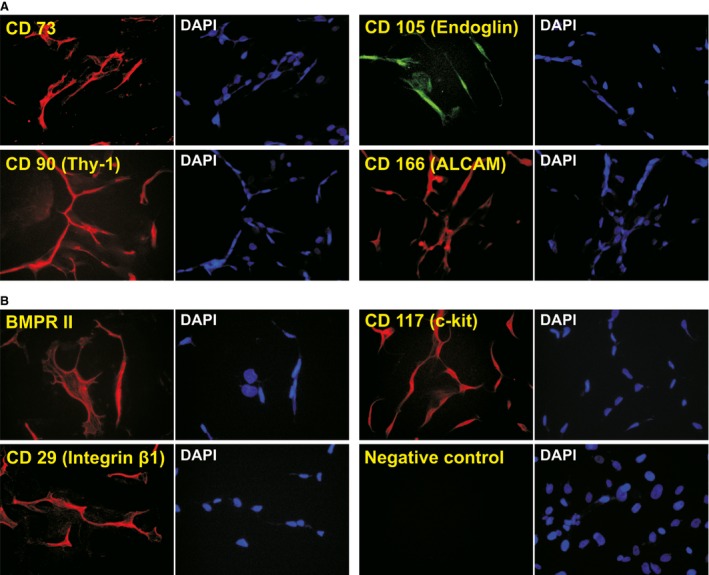
The iSuPs express most MSC markers. The iSuPs were seeded at subconfluence and stained with antibodies against the MSC consensus markers (CD73, CD105/endoglin, CD90/Thy‐1, and CD166/ALCAM) (**A**), and other MSC/progenitor markers (BMPR‐II, CD117/c‐kit or CD29/Integrin β1)(**B**), as reported 43, 56. Stains without primary antibodies were used as negative controls. Cell nuclei were counter‐stained with DAPI. Representative images are shown.

It has been reported that cranial suture tissues contain MSC‐like progenitor cells 6, 8, 10, 66. We examined whether the immortalization process would affect the expression of MSC markers. It has been reported that consensus human MSC markers include CD73, CD105/endoglin, CD90/Thy‐1, CD166/ALCAM 67. Using immunofluorescence staining, we detected whether the iSuPs express these MSC markers. We found that all of these consensus markers were readily detectable in the majority of the iSuPs (Fig. 2A). Moreover, the expression of other MSC and/or progenitor markers also has been detected, such as BMPR‐II, CD117/c‐kit and CD29/Integrin β1 (Fig. 2B). Collectively, these results demonstrated that iSuPs express most of the consensus MSC markers, suggesting that these cells may possess MSC‐like characteristics.

### The iSuPs are capable of differentiating into osteogenic and adipogenic lineages upon BMP9 stimulation

**Figure 3:**
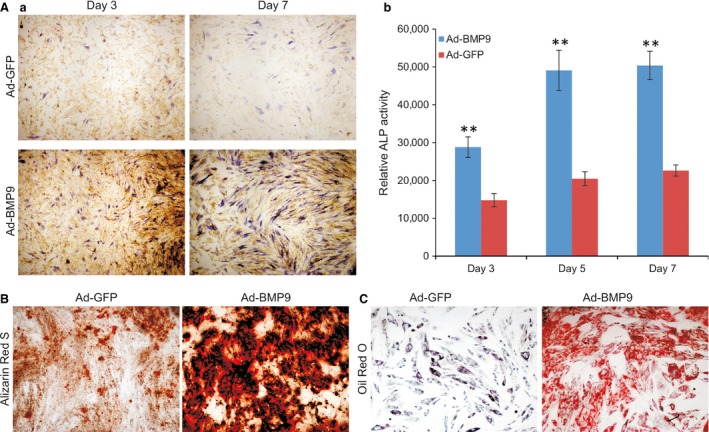
BMP9 induces osteogenic and adipogenic differentiation in iSuPs *in vitro*. (**A**) The activity of early osteogenic marker alkaline phosphatase (ALP) is efficiently induced by BMP9 in iSuPs. Subconfluent iSuPs were infected with Ad‐BMP9 or Ad‐GFP. Cells were either fixed and histochemically stained for ALP activity (*a*) or subjected to ALP quantitative analysis at the indicated time‐points (*b*). Each assay condition was performed in triplicate. ‘**’*P* < 0.01. (**B**) Matrix mineralization evaluated by Alizarin Red S staining. Ad‐BMP9‐ or Ad‐GFP‐infected iSuPs were maintained in mineralization medium for 14 days and stained with Alizarin Red S. Each assay condition was performed in triplicate. Representative images are shown. (**C**) Adipogenic differentiation evaluated by Oil Red O staining. Ad‐BMP9‐ or Ad‐GFP‐infected iSuPs were maintained for 10 days and subsequently subjected to Oil Red O staining. Each assay condition was performed in triplicate. Representative images are shown.

We previously demonstrated that that BMP9 is one of most osteogenic BMPs among the human BMP family and can induce osteogenic, adipogenic and, to a lesser extent, chondrogenic differentiation 32, 33, 34, 35. We analysed the osteogenic potential of the iSuPs *in vitro*. When the early osteogenic marker ALP was evaluated, BMP9 was shown to induce a stronger ALP staining in the iSuPs than that of control group at the indicated time‐points although basal ALP activity was also detected at as early as day 3 (Fig. 3A, panel a). The ALP activity induced by BMP9 in the iSuPs was further assessed quantitatively, and similar results were obtained (Fig. 3A, panel b). We also conducted Alizarin Red S staining to assess matrix mineralization, which is an indicator of the late stage of osteogenic differentiation *in vitro*. When BMP9‐infected iSuPs were cultured in mineralization medium for 14 days, apparent mineralized extracellular matrix nodules were observed (Fig. 3B). Collectively, the above results indicated that the iSuPs are responsive to BMP9 and retain osteogenic potential upon BMP9 stimulation.

We further examined the adipogenic potential of the iSuPs. When iSuPs were transduced with BMP9 for 10 days, Oil Red O staining revealed that the cells exhibited a significantly higher level of lipid droplet formation than that of the control group (Fig. 3C). These results strongly suggest that the iSuPs may possess adipogenic potential upon BMP9 stimulation, and that iSuPs may be multipotent and capable of differentiating into different MSC lineages upon BMP9 stimulation.

### FLP recombinase‐mediated removal of SV40 T antigen decreases the proliferative activity and increases endogenous osteogenic and adipogenic capability of the iSuPs

**Figure 4:**
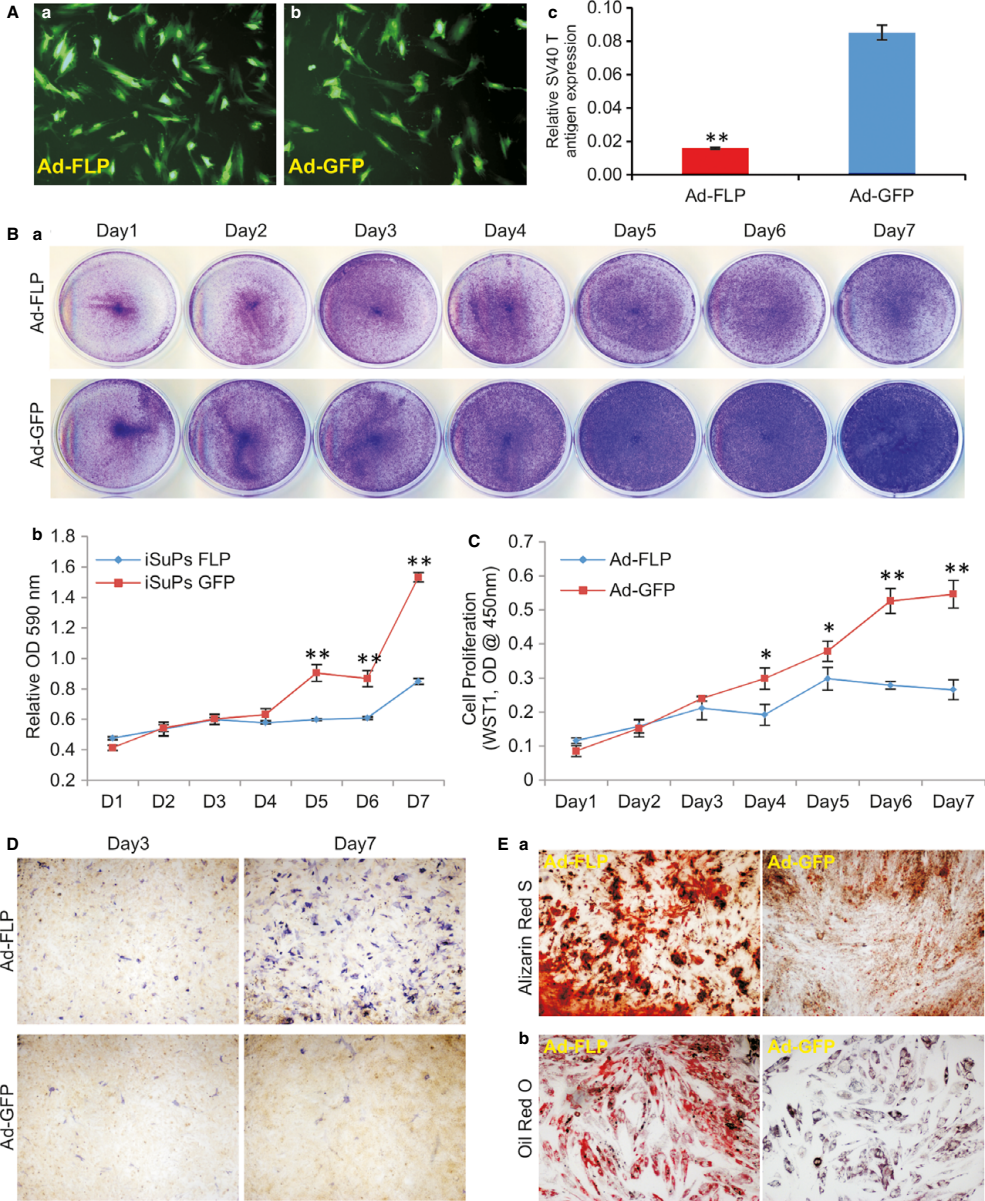
FLP recombinase‐mediated removal of SV40 T antigen decreases the proliferative activity and increases the endogenous osteogenic and adipogenic activities of iSuPs. (**A**) Efficient removal of SV40 T antigen from iSuPs by Ad‐FLP. Subconfluent iSuPs were infected with Ad‐FLP (*a*) or Ad‐GFP (*b*). At 3 days after infection, total RNA was isolated and subjected to RT‐PCR and subsequently TqPCR analysis of SV40 T antigen expression. GAPDH served as a reference gene. ‘**’*P* < 0.001. (**B**) Cell proliferation assay assessed by crystal violet staining. Ad‐FLP or Ad‐GFP‐infected iSuPs were fixed for crystal violet staining at indicated time‐points (*a*) and the stained cells were dissolved and quantitatively measured at A_590 nm_ (*b*). Each assay condition was performed in triplicate. Representative results are shown. ‘**’*P* < 0.001. (**C**) Cell proliferation by WST‐1 analysis. Subconfluent iSuPs were infected with Ad‐FLP or Ad‐GFP and seeded in 96‐well plates. WST‐1 substrate was added to the cell culture medium and A_450 nm_ readings were recorded at the indicated time‐points. Assays were performed in triplicate. ‘*’*P* < 0.05; ‘**’*P* < 0.001. (**D**) Effect of the SV40 T antigen removal on ALP activity in iSuPs. Subconfluent iSuPs were infected with Ad‐FLP or Ad‐GFP and subsequently stained for ALP activity at the indicated time‐points. (**E**) The effect of FLP‐mediated reversal on matrix mineralization and adipogenesis of iSuPs. (*a*) Alizarin Red staining: Subconfluent iSuPs were infected with Ad‐FLP or Ad‐GFP and cultured in mineralization medium. At 14 days after infection, cells were subjected to Alizarin Red S staining. (*b*) Oil Red O staining: Subconfluent iSuPs were infected with Ad‐FLP or Ad‐GFP and cultured for 10 days, followed by Oil Red O staining. Representative images are shown.

As shown in Figure 1B, the immortalizing gene SV40 T antigen can be excised *via* the action of FLP recombinase on the flanking FRT sites. We tested whether the immortalization could be effectively reversed in iSuPs. A recombinant adenoviral vector Ad‐FLP has been used to effectively express FLP 46, 56. We found that the iSuPs could be infected by Ad‐FLP or Ad‐GFP with a high efficiency (Fig. 4A, panels a & b). The efficient removal of SV40 T antigen by FLP was confirmed by TqPCR analysis in Ad‐FLP‐infected iSuPs, but not in the GFP control group (Fig. 4A, panel c). The cell proliferation rate of the Ad‐FLP‐infected iSuPs was decreased as assessed by crystal violet staining (Fig. 4B, panel a), while quantitative analysis of the staining showed that the decrease in cell growth was obviously lower from day 5 to day 7 than that of the GFP group (*P* < 0.001) (Fig. 4B, panel b). Furthermore, WST‐1 cell proliferation revealed that the Ad‐FLP‐transduced iSuPs had a lower proliferation rate, at as early as day 4 after infection, than that of control group (Fig. 4C). Collectively, these results strongly suggest that the immortalization of iSuPs may be effectively reversed by FLP recombinase.

We further analysed the spontaneous differentiation potential of the iSuPs upon Ad‐FLP‐mediated removal of SV40 T antigen in iSuPs. We found that when iSuPs were infected with Ad‐FLP or Ad‐GFP, the ALP activity increased in the Ad‐FLP‐infected iSuPs, compared to that of the Ad‐GFP‐infected control group (Fig. 4D). The effect of FLP‐mediated reversal on late stage differentiation of the iSuPs was also examined. When the iSuPs were infected with Ad‐FLP or Ad‐GFP and cultured in mineralization medium for 14 days, we found that FLP‐infected iSuPs had a significantly greater amount of matrix mineralization nodules, as assessed by Alizarin Red S staining, than that of the GFP‐infected group (Fig. 4E, panel a). Furthermore, when the iSuPs were transduced with Ad‐FLP or Ad‐GFP for 10 days and stained with Oil Red O staining, we found that the FLP‐infected iSuPs were able to undergo adipogenic differentiation, compared with that of GFP‐infected control iSuPs (Fig. 4E, panel b). Thus, these results indicate that FLP‐transduced iSuPs are able to undergo spontaneous differentiation into osteogenic and adipogenic lineages.

### BMP9 regulates the expression of multiple lineage regulators and the craniosynostosis‐associated gene Nell‐1 in iSuPs

**Figure 5:**
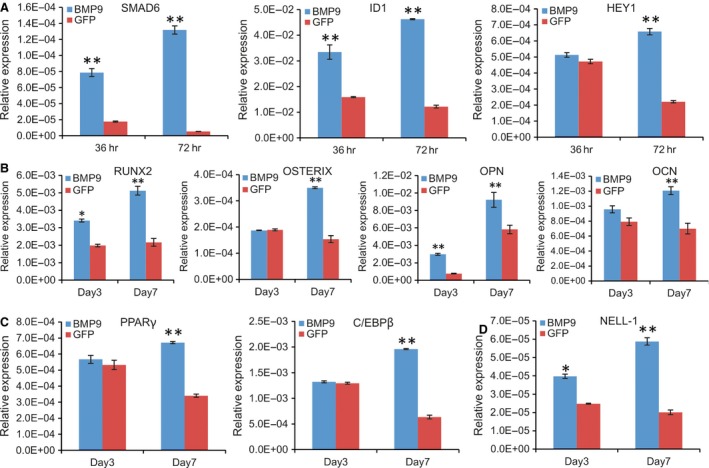
BMP9 regulates the expression of downstream target genes and osteogenic and adipogenic lineage regulators in iSuPs. Subconfluent iSuPs were infected with Ad‐BMP9 or Ad‐GFP. Total RNA was isolated at the indicated time‐points and subjected to TqPCR analysis for the expression of the known BMP9‐induced target genes (**A**), osteogenic regulators and late markers (**B**), adipogenic regulators (**C**) and the craniosynostosis‐associated gene NELL‐1 (**D**). All samples were normalized with GAPDH expression levels. TqPCR reactions were performed in triplicate. Relative expression was calculated by dividing the qPCR value of a given gene of interested with its respective GAPDH value. ‘*’*P* < 0.05; ‘**’*P* < 0.01.

We previously identified several early key downstream mediators of BMP9 signalling in MSCs, such as Smad6, Id1 and Hey1 68, 69, 70. Here, we tested whether the iSuPs were responsive to BMP9 stimulation. When the iSuPs were infected with Ad‐BMP9 or Ad‐GFP for 36 and 72 hrs, TqPCR analysis showed that both Smad6 and Id1 were significantly up‐regulated in BMP9‐infected iSuPs at 36 or 72 hrs, while the expression of Hey1 was significantly up‐regulated at 72 hrs, compared with that of the Ad‐GFP‐infected group (*P* < 0.01) (Fig. 5A), indicating that the iSuPs are responsive to the stimulation of BMP9.

Given the fact that iSuPs express most of MSC markers, we next tested whether the iSuPs were able to differentiate into osteogenic, chondrogenic and adipogenic lineages. When the iSuPs were infected with Ad‐BMP9, Runx2 and Osterix, both osteogenic lineage regulators, were remarkably up‐regulated at day 7, while the expression of the late osteogenic markers osteocalcin and osteopontin was also significantly increased at day 7 (Fig. 5B). We further analysed the expression of adipogenic lineage regulator PPARγ and the early adipogenic marker C/EBPβ 35, 71, and both genes were significantly up‐regulated at day 7 (Fig. 5C). Interestingly, the chondrogenic regulator Sox9 was up‐regulated in the iSuPs by BMP9 at day 7, but the change did not reach any statistical significance (data not shown). This is not surprising considering the fact that the iSuPs were derived from cranial sutures, which are formed through intramembranous ossification rather than endochondral ossification.

We further examined whether Nell‐1 was regulated by BMP9 in the iSuPs. Nel‐like molecule‐1 (Nell‐1) is a secreted protein first identified through its overexpression in pathologically fusing and fused suture specimens from patients with craniosynostosis. Because that either overexpression or deficiency of Nell‐1 could lead to significant cranial abnormalities in mice 72, 73, we tested whether BMP9 induced the expression of Nell‐1 in the iSuPs. When the iSuPs were infected with Ad‐BMP9 or Ad‐GFP, quantitative TqPCR analysis showed that Nell‐1 was significantly up‐regulated in BMP9‐infected iSuPs at as early as day 3 (*P* < 0.05) and continued to increase at day 7 (Fig. 5D). These results indicate that Nell‐1 may be regulated by BMP9 in the iSuPs.

### BMP9 induces effective osteogenesis and adipogenesis of iSuPs in a stem cell implantation model *in vivo*

**Figure 6:**
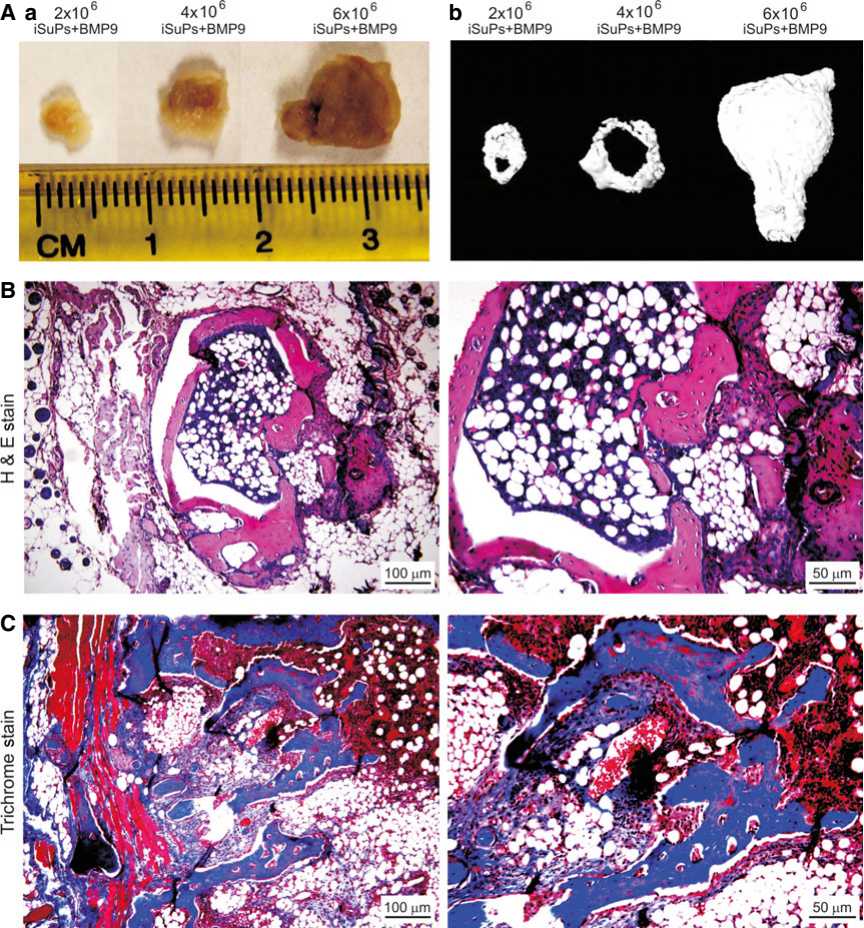
BMP9 effectively induces osteogenesis and adipogenesis from the iSuPs *in vivo*. Subconfluent iSuPs were infected with Ad‐BMP9 or Ad‐GFP for 24 hrs, collected and resuspended in PBS for subcutaneous injection into the athymic nude mice at three doses: 2 × 10^6^ cells/injection, 4 × 10^6^ cells/injection and 6 × 10^6^ cells/injection (*n* = 8 each group). At 4 weeks after implantation, masses formed at the injection sites were retrieved. No detectable masses were formed in the Ad‐GFP‐infected iSuPs group. (**A**) Macrographic images (*a*) and micro‐CT imaging (*b*) of the retrieved masses. Representative results are shown. (**B**) Histologic analysis. The retrieved masses were fixed and decalcified and subjected to H & E staining. Representative images of the low and high‐power magnifications are shown. (**C**) Trichrome staining. Sections of the retrieved and fixed samples were subjected to trichrome staining. Representative images of the low and high‐power magnifications are shown.

We further tested whether the iSuPs were to undergo multilineage differentiation *in vivo* using the well‐established stem cell implantation assay 46, 74. The iSuPs infected with Ad‐BMP9 or Ad‐GFP were subcutaneously injected into the flanks of athymic nude mice with three different dosages. At 4 weeks after injection, the masses were retrieved from the mice injected with BMP9‐infected iSuPs, and we found the sizes of the masses were roughly proportional to the numbers of Ad‐BMP9‐transduced iSuPs injected (Fig. 6A, panel a). The bony masses were further confirmed by μCT imaging (Fig. 6A, panel b). No detectable masses were formed in the Ad‐GFP group (data not shown). In fact, the injection sites of the iSuPs infected with Ad‐GFP disappeared in approximately 10 days after injection. Histologic analysis of the retrieved masses indicated that the BMP9‐transduced iSuPs formed mature and highly mineralized osteoid matrix, while densely populated adipocytes were also readily detected (Fig. 6B). Trichrome staining further confirmed that the masses retrieved from the BMP9‐infected iSuPs exhibited highly mature and well‐mineralized trabecular bony structure (Fig. 6C). Therefore, these *in vivo* results strongly indicate that the iSuPs are multipotent progenitor cells and have osteogenic and adipogenic potential, which can be effectively induced by BMP9.

## Discussion

Although MSCs can be isolated from various tissues including bone marrow, periosteum, liver, adipose, brain, skeletal muscle and amniotic fluid 21, 22, 23, 24, they exhibit significant differences in their expression profiles and differentiation potential 75. There are three minimal criteria to define human MSCs 67. First, MSCs must be plastic‐adherent when maintained in standard culture conditions. Second, MSCs must express specific surface antigen such as CD105, CD73 and CD90. Third, MSCs must differentiate to osteoblasts, adipocytes and chondroblasts *in vitro* 67.

It has been reported that MSCs can be derived from cranial bone marrow and cranial suture mesenchyme 6, 8, 20, 25. However, the human primary cranial suture cells have limited life span *in vitro* and may lose osteoblastic potential after certain numbers of passages 19. Thus, there is an essential need to establish stable human cranial suture stem cells (SuPs). Using a reversible SV40 T antigen‐based immortalization system, we have demonstrated that primary suture stem cells retrieved from the patent sutures of craniosynostosis patients can be efficiently immortalized. The iSuPs maintain long‐term proliferative activity and express most of the MSC markers. The iSuPs are multipotent as they can differentiate into osteogenic and adipogenic lineages upon BMP9 stimulation both *in vitro* and *in vivo*. Furthermore, using the ectopic bone formation model, we found that primary SuPs exhibited similar osteogenic and adipogenic capabilities to that of the iSuPs upon BMP9 stimulation (data not shown), indicating that SV40 T antigen‐mediated immortalization does not significantly impact the lineage commitment and differentiation potentials of primary suture progenitor cells. However, primary SuPs have a limited life span in culture. Thus, the iSuPs should serve as a valuable tool to study the molecular mechanism of craniosynostosis, as well as to study the process of intramembranous ossification.

In this study, we also find that BMP9 up‐regulates the expression of Nell‐1 in the iSuPs. Nell‐1 was initially detected at fusing and fused sutures from craniosynostosis patients 76. Nell‐1 is an osteoinductive growth factor 76, 77. It has been shown that either overexpression or deficiency of Nell‐1 leads to significant cranial abnormalities in mice 72, 73. The expression of Nell‐1 is tightly regulated by Runx2, a vital mechanistic convergence point for the development of craniosynostosis 77. Thus, it is conceivable that this up‐regulation of Nell‐1 may be resulted from the BMP9‐induced up‐regulation of Runx2, suggesting that BMP9 may play a potential role in regulating the patency of cranial sutures and the development of craniosynostosis.

While we demonstrate that the iSuPs express most of the MSC markers and are osteogenic and adipogenic, we fail to detect apparent chondrogenic potential of the iSuPs both *in vitro* and *in vivo* upon BMP9 stimulation. This may be attributed to the fact that the iSuPs are derived from cranial sutures, where intramembranous ossification, but not endochondral ossification, is the dominant process of bone formation. Thus, the iSuPs may be used as valuable MSC‐like progenitors to study the underlying mechanism of intramembranous ossification. Nonetheless, we also demonstrate that the primary SuPs are proliferative and highly osteogenic and thus may be used as a potential source of MSC progenitors for cranial bone engineering.

Using the SV40 T antigen, one of the most commonly used immortalization genes, we have successfully immortalized numerous cell lines, including embryonic fibroblasts 43, 56, adipose‐derived MSCs (iMADs) 46, stem cells from dental apical papilla 78, mouse melanocytes 44, hepatic progenitor cells 41, 42, 79, calvarial mesenchymal progenitor cells 80, articular chondrocytes 47, foetal heart progenitors 45 and mouse Achilles tenocytes 48. In this study, we utilized SV40 T antigen and successfully established the human iSuPs. However, based on our experience, it is relatively less efficient to immortalize human progenitor cells using SV40 T antigen alone.

Traditional cell immortalization and transformation strategies usually involve in overexpression of oncogenes and/or inactivation of tumour suppressor genes 81. The commonly used oncogenes include telomerase (TERT), KRAS, c‐MYC, CDK4, while the frequently inactivated tumour suppressor genes are p53, Rb and p16^INK^. It is widely accepted that the ability of SV40 T antigen to immortalize cells is mediated by its ability to complex with p53 and inhibit p53 functions although the exact molecular mechanisms underlying SV40 T antigen‐induced immortalization remain to be fully understood. Nonetheless, unlike the above‐mentioned oncogenes, SV40 T antigen‐transformed cells are usually not tumorigenic 82, 83, which is confirmed in our *in vivo* studies with the iSuP cells.

In summary, to overcome the technical challenges in maintaining sufficient and long‐term culture of SuPs for suture biology studies and/or possible clinical therapies, we demonstrate that SuPs can be efficiently immortalized by SV40 T antigen. The reversibly immortalized iSuPs exhibit high proliferative activity and retain long‐term cell proliferation, which can be reversed by introducing FLP recombinase. The iSuPs express most of the MSC markers and retain osteogenic and adipogenic differentiation potential upon BMP9 stimulation both *in vitro* and *in vivo*. Thus, the iSuPs should be a valuable resource to develop novel strategies of molecular therapy in craniosynostosis or cranial deficiency, as well as to study the processes of intramembranous ossification and adipogenesis. Furthermore, BMP9 may be further explored as a novel factor for cranial bone engineering.

## Conflicts of interest

All authors declare no financial conflict of interests.

## Author's contributions

TCH, DH, RRR, MJL, JMW and DS designed the project; TCH, DH, RRR, MJL, JMW and DS wrote the manuscript; DS, JY and FZ performed most of the experiments; QW, JLiao, YZ, JF, CM, XH, XQ, LC, LL, YY, XY and ZZhang provided assistance in some experiments and helped on data collections and analyses; CZ, ZZeng, RZ, SY, TW, XW, YS, JLei, YL and WZ provided materials/reagents for the project and assisted some of the experiments; all authors, read and approved the manuscript.

## Supporting information


**Table S1** List of TqPCR Primers.
